# Identification of miR-24 and miR-137 as novel candidate multiple sclerosis miRNA biomarkers using multi-staged data analysis protocol

**DOI:** 10.22099/mbrc.2017.24861.1256

**Published:** 2017-09

**Authors:** Farveh Ehya, Hossein Abdul Tehrani, Masoud Garshasbi, Seyed Masood Nabavi

**Affiliations:** 1 Department of Medical Biotechnology, Faculty of Medical Sciences, Tarbiat Modares University, Tehran, Iran; 2 Department of Medical Genetics, Faculty of Medical Sciences, Tarbiat Modares University, Tehran, Iran; 3 Department of Neurology, Mostafa Hospital, Shahed University, Tehran, Iran; 4 Department of Regenerative Biomedicine, Cell Science Research Center, Royan Institute for Stem Cell Biology and Technology, ACECR, Tehran, Iran

**Keywords:** miRNA-mRNA regulatory network, Data integration, miR-24, miR-137

## Abstract

Many studies have investigated misregulation of miRNAs relevant to multiple sclerosis (MS) pathogenesis. Abnormal miRNAs can be used both as candidate biomarker for MS diagnosis and understanding the disease miRNA-mRNA regulatory network. In this comprehensive study, misregulated miRNAs related to MS were collected from existing literature, databases and via in silico prediction. A multi-staged data integration strategy (including the construction of miRNA-mRNA regulatory network and systematic data analysis) was conducted in order to investigate MS related miRNAs and their regulatory networks. The final outcome was a bi-layer MS related regulatory network constructed with 27 miRNAs (seven of them were novel) and 59 mRNA targets. To verify the accuracy of the bioinformatics strategy three novel and five previously reported miRNAs from the network model were selected for experimental validation using the real-time PCR assay. The obtained results proved the accuracy of the network. The expression of themiR-24 and miR-137(as novel MS candidate biomarker) and miR-16, and miR-181 (as previously reported MS candidate biomarker) showed significant deregulation in 33 MS patients compared to the control. The optimized data integration strategy conducted in this study found two miRNAs (miR-24and miR-16)that can be considered as candidate biomarkers for MS and also has the potential to generate a regulatory network to aid in further understanding the mechanisms underlying this disease.

## INTRODUCTION

Multiple sclerosis (MS) is a chronic inflammatory disease involving the demyelination of central nervous system, with three main clinical subtypes: relapsing-remitting (RRMS), primary and secondary progressive (PPMS & SPMS). Current protocol for diagnosis of MS involves MRI scans backed up with lumber puncture. In recent years, many of research done has focused on the quantification of circulating micro RNAs (miRNAs) in peripheral blood of the MS patients as a noninvasive alternative diagnostic protocol. This could prove to be less aggressive than current methods, in turn allowing for more frequent and accessible testing and hence early detection of the disease. In MS, dysregulation of miRNAs can be seen and it has been shown that MS can trigger the activation of myelin specific T lymphocytes. Abundant miRNAs expression either in immune cells (disease mediator) or central nervous system (CNS, disease target organ) has been reported and lots of evidence confirms their role in MS pathogenesis [1].

MiRNA profiling in application to find MS miRNA biomarkers have been done using different cell populations of MS patients (blood cells, brain, CNS lesions) and only some studies have been performed based on cell-free circulating miRNAs. Since a vast number of studies focused on the field of MS biomarker discovery, a comprehensive bioinformatic analysis could be helpful to getting all the robust data together. For example in one study miR-149-3p has been reported as a deregulated miRNA in MS [2]and another report has indicated that miR-150 could be a candidate biomarker for MS [3]. Another problem is the nature of high throughput profiling of miRNAs; although this technique is very robust, the rigidity of data analysis can omit miRNAs that are specific but low abundance. For example, miR-155 and miR-145 have been reported as candidate MS biomarkers byeight [4-11] and five [9, 12-15] independent studies respectively. However, it has been confirmed that these two miRNAs are housekeeping-like miRNAs and regulate different pathological and physiological processes. Therefore, one can argue whether these reported miRNAs are specific enough at all to be considered as robust biomarkers [16]. 

Currently, with the development of microarray and high-throughput sequencing technologies, more molecular data and multi-omic information are generated across multiple data types. New explorations in omic data led to the development of many new methods for *data integration*. It generally means the incorporation of multi-omic information in a meaningful way to provide a more comprehensive analysis of a biological point of interest. Data integration can provide increased analysis power in order to identify the important genomic factors and their interactions, in turn furthering our understanding of complex biological processes [17].

In this study, we propose a multi-staged integrative method of data analysis which is a stepwise analysis method that reduces the search space through different stages of analysis, followed by systematic analysis, allowing more accurate and effective outcomes [17]. The main purpose of our study is to find potential miRNAs regulating pathways involved in the pathogenesis of MS disease and to construct their regulatory network. These miRNAs may be used as candidate biomarkers for MS diagnosis and help to understand the relationship between circulating miRNAs and the pathogenicity of the disease.

## MATERIALS AND METHODS

**Figure 1:**
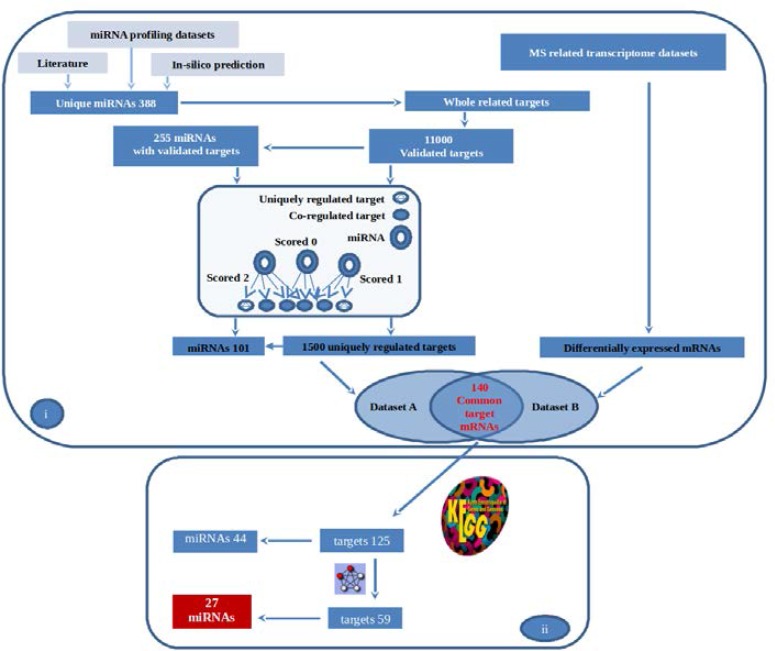
Schematic workflow for the identification of potential MS miRNA biomarkers. Data collected and processed in two parts: (i) data integration approach and (ii) systematic analysis

**Figure 2:**
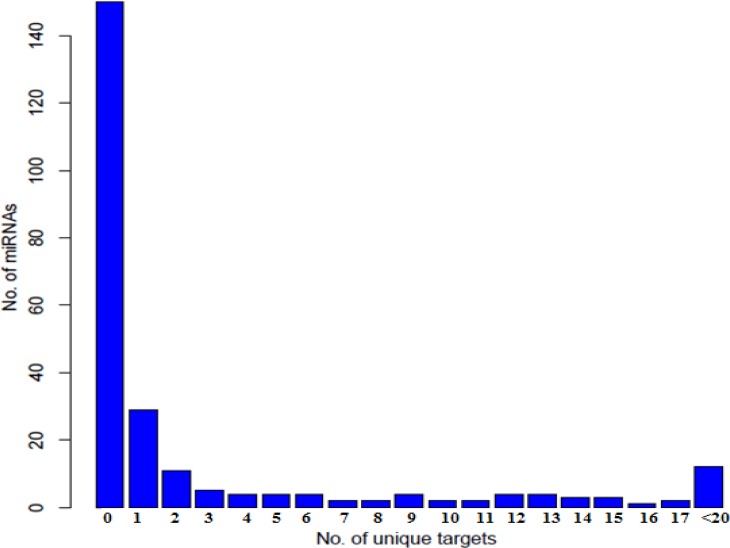
Distribution of the MS related miRNAs against different numbers of unique targets. All MS related miRNAs were scored based on the number of their uniquely regulated targets

**Data collection and miRNA-mRNA regulatory network construction: **In this study we developed a bioinformatics protocol that involves combining multi-staged data integration approaches with systematic analysis in order to construct a miRNA-mRNA regulatory network for MS. Data integration relies on the combination of samples from different molecular type in order to identify groups of objects that share relevant molecular characteristics. Omics datasets are essential in data integration approaches. For this purpose, we collected and processed data using the strategy outlined in Figure 1. First, we collected all misregulated miRNAs reported in MS related research papers and noncoding RNA profiling datasets. The resulting data was expanded by adding in silico predicted miRNAs based on available MS related genes and pathways. To process the collected data more specifically, we searched for the experimentally validated targets of collected miRNAs and used them to construct a primary miRNA-mRNA regulatory network. The resulting miRNAs from the network were scored based on the number of their uniquely regulated targets and miRNAs with co-regulated targets (zero scored miRNAs) were removed (Fig. 2).The remaining targets were then compared with the misregulated mRNAs retrieved from MS related transcriptome datasets and the common mRNAs (and subsequently their relevant miRNAs) were selected. The selected mRNAs were checked against KEGG pathway database in order to identify significant pathways. These were applied on S database for protein-protein interaction analysis. The final outcome was 27 mi RNAs and 59 mRNA which were used to construct a miRNA-mRNA regulatory network.

**Data** **collection: **Data was collected from three separate sources; literature, gene expression omnibus (GEO, http//www.ncbi.nlm.nih.gov/GEO) database and in silico generated predictions. The method of data mining for each of these sources is described bellow in more detail. Relevant research papers in the field of MS biomarker discovery (high and low throughput studies) were searched using query keywords of "MS and miRNA", "MS and miRNA biomarker", "multiple sclerosis and miRNA" and literatures deposited in miRWalk (http://www.ummm.uni-heidelberg.de) were also added to these search results. The database, GEO was searched for MS-related miRNA profiling of peripheral blood samples. Six datasets were selected and their normalized data were downloaded directly. MiRNAs were filtered according to false discovery rate (FDR) adjusted p-values ≤ 0.05 (cut off). For in silico prediction, genes and pathways that were reported to be involved in MS pathogenesis according to NCBI/Gene (http://www.ncbi.nlm.nih.gov/gene),miRecords (http://miRecords.umn.edu/miRecords),Tarbase(http://www.diana.pcbi.upenn.edu/tarbase), HOCTARdb (http://hoctar.tigem.it/) were picked out and their relevant miRNAs were selected.The resulting datasets from all three sources were combined with the removal of any repeating miRNAs. For transcriptome collection, preprocessed data of six MS related transcriptome datasets were retrieved from GEO, three of which were relevant to previously selected miRNA datasets. All mRNAs were filtered according to the cut off value (adjusted p-values ≤ 0.05). Analysis was performed using R version 2.14.1.

**Construction of miRNA-mRNA** **regulatory** **network: **For construction of miRNA-mRNA network, the collected miRNA data was searched against several databases such as PicTar (http://pictar.mdc-berlin.de/) and TargetScan (http://www. targetscan.org/) to find their targets. Since experimentally validated miRNA-mRNA relationship is important for identification of MS related miRNAs biomarkers, validated targets were selected and used to construct the miRNA-mRNA network. 

**Systematic analysis: **KEGG pathway database[18]were searched for the target genes. Protein-protein interaction was considered in STRING 10 database (http://string-db.org/) for corresponding proteins of the genes with valid KEGG pathways.

**Experimental validation of** **bioinformatic** **results Sample collection: **Thirty three wash-out and naive Iranian MS patients from all three clinical subtypes of MS who were diagnosed based on McDonald's criteria [19] by a neurologist (Table 1) and 30 Iranian healthy individuals (control group)were selected and written informed consent was obtained before blood sampling. Sample collection was done under the supervision of the neurologist at Mostafa Khomeini Hospital. This study was approved by the Ethics Committee of Tarbiat Modares University. Red cap Vacutainer tubes were used for blood collection. Serum samples were isolated in accordance with the standard serum separation protocol [20] and immediately transferred to -80^o^C for future analysis.

**Table 1:** Summary of MS patients' information

	**Control**	**RRMS**	**SPMS**	**PPMS**
**Total number of samples**	30	16	11	6
**Female/Male**	26/4	16/0	10/1	5/1
**Age in years, (range), median**	(22-57), 28	(14-45), 25	(25-42), 32	(36-55), 40
**Disease duration (onset-to -sampling) (range), mean, SD**	-	(1M-24Y), (8,7.23)	(3Y-20Y), (12, 4.96)	(0-20Y),( 8, 8.17)
**EDSS, (range), mean, SD**	-	(0-6.5), (1.68,2.86)	(4-7.5), (6.2, 2.9)	(0-7.5), (4.6,2.65)

**RNA isolation and real-time PCR assay: **Total RNAs were isolated from 300 µl of serum samples using miTotal RNA mini kit (Viogene, Vietnam) in accordance with the manufacturer's instructions and their quality and quantity were checked by UV-visible spectrophotometer (Thermo Scientific). Universal enzymatic ploy (A) polymerase tailing was performed for miRNAs. Primers were designed based on miRNA sequences deposited in miRBase database (version 20, June 2014) (http://www.mirbase.org/) with Primer3 design program (http://primer3.sourceforge. net/). cDNA was synthesized using poly (A) tailed miRNAs and cDNA Synthesis Kit (ParsGenome, Iran) in accordance with the manufacture's instruction; In brief 2 µl of 5X reaction buffer, 10 mM of dNTP mix, 0.5 µl RT enzyme, 15 pmol miR cDNA Syn specific primer and 5 µl poly (A) tailed miRNAs were mixed together in a reaction volume of 10 µl.The reaction was incubated at 45^o^C for 60 minutes followed by 1 minute at 85^o^C. Real time PCR was performed in triplicate with the Rotor-Gene RG-3000 and each reaction contained 1 µl (5 time diluted) cDNA and 10 µl of SYBR premix Ex Taq (TaKaRa, Bio Inc. Japan) and 10 pmol of mix of miRNA specific primers with total reaction volume of 20 µl. All quantitative PCR values were normalized to those of U6 [21] and the results were analyzed based on 2^-ΔΔCt^method [ 22]. All statistical analysis was done in R version 3.1.1.

## RESULTS

In order to identify MS related miRNAs, data were collected from three different sources and bioinformatically processed as shown in the schematic workflow represented in Fig. 1.

**Figure 3:**
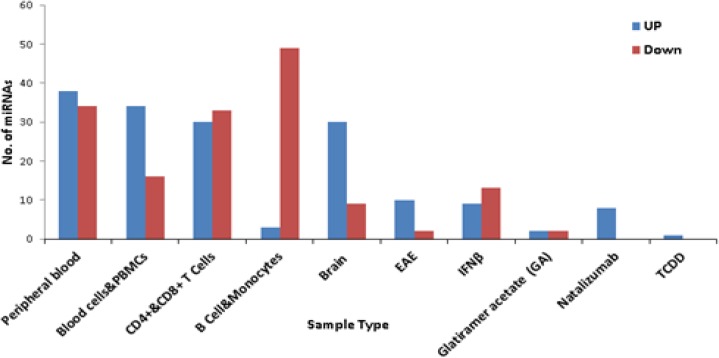
The frequency of the differentially expressed miRNAs in MS disease. The data were collected from 23 selected research papers. All MS related miRNAs were separated by their origins or the type of drug treatments

To find miRNAs as MS candidate biomarkers, all available papers that revealed the role of miRNAs in MS pathogenicity were searched as previously mentioned in method section 1.1 and relevant research papers were obtained. From these,240 differentially expressed miRNAs were selected, 125 were up regulated and 115 down regulated. Figure 3 shows the differentially expressed miRNAs and the sources they have been taken from (Supplementary Table S1). The maximum numbers of differentially expressed miRNAs are found from those studied that have been conducted on the peripheral blood of MS patients. In order to find novel MS related miRNAs, noncoding RNA profiling datasets deposited in GEO were searched and 6 datasets were selected. Out of these datasets, 259 unique and misregulated miRNAs were obtained (p<0.05, Supplementary Table S2). For the in silico part, 55 miRNAs which may target MS related genes and 26 miRNAs which may regulate MS related pathways were predicted (Supplementary Table S3, S4).Finally, after combining these three datasets with redundancy removed, 388 unique miRNAs were obtained(Supplementary Table S5).

The targets of 388 unique miRNAs were searched as mentioned in method section 1.2 and only 255 miRNAs which they had validated targets (11000 targets) were selected. Previously it has been hypothesized that if one miRNA has more uniquely regulated targets it has the potential to be a more specific biomarker [23]; Based on this criterion, each miRNA was scored based on the number of its uniquely regulated targets; in other word all miRNAs with co-regulated targets (or a score of zero) were removed (Fig. 2).This left us with 101 miRNAs with nearly 1500 out of a total of 11000 validated targets. The 1500uniquely regulated targets were considered as dataset A. In order to find the most specific targets for each of the remaining miRNAs, all MS related transcriptome profiling datasets deposited in GEO database were searched and differentially expressed mRNAs were selected and grouped as dataset B (Supplementary table S2). 

**Figure 4:**
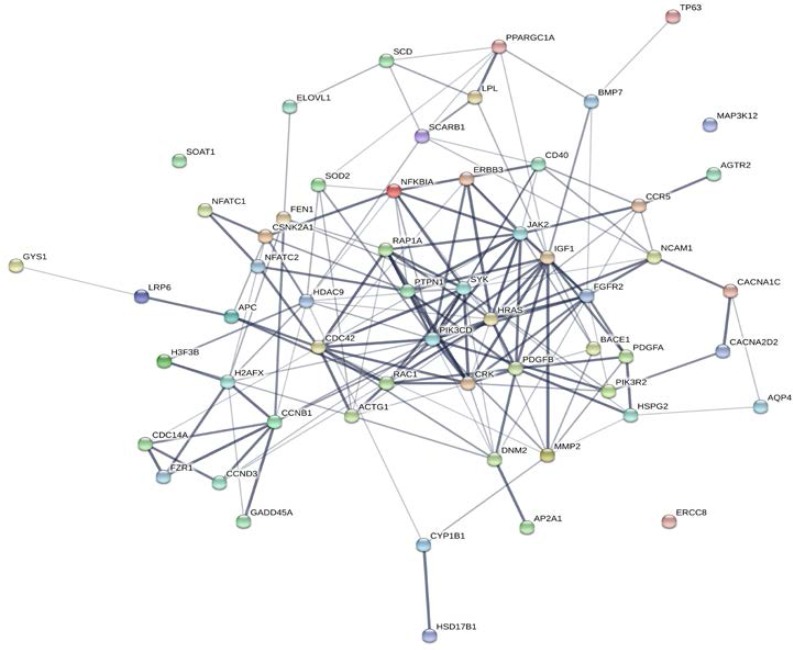
Protein- protein interaction (PPI) network. The most significant PPIs involved in MS based on the final result were shown (p-value <0.05). The data was extracted from the KEGG pathway and generated with STRING 10.0. Each node represents one protein. Line thickness indicates the strength of data support

By comparing datasets A and B, 140 mRNAs (targets) were found to be common between both datasets. These targets are valid and it has been shown that their expressions are significantly deregulated in MS. The corresponding genes for these mRNAs were looked up in pathways database in order to find their roles using the protocol mentioned in the methods section 1.3. Out of these 140 validated targets, 125 genes (belonging to 44 miRNAs) had valid KEGG annotation and the most enriched KEGG pathways were axon guidance, transcriptional misregulation in cancer, and cytokine-cytokine interaction (Supplementary **table S6**). All 125 remaining targets were surveyed for their protein-protein interactions using STRING database and 59 of them were found to show the most significant co-interactions (Fig. 4).They were uniquely regulated by 27 miRNAs out of 101 remaining ones. Therefore, the result of our bioinformatics refining strategy found a network comprising 27 miRNAs and 59 targets (Table 2 and Fig. 4, 7). Seven miRNAs out of 27 miRNAs (miR-24, miR10a, miR-137, let-7a, let-7e, miR-216, miR-330) are novel and have not been reported as candidate MS biomarkers (as of December 2015).

**Figure 5:**
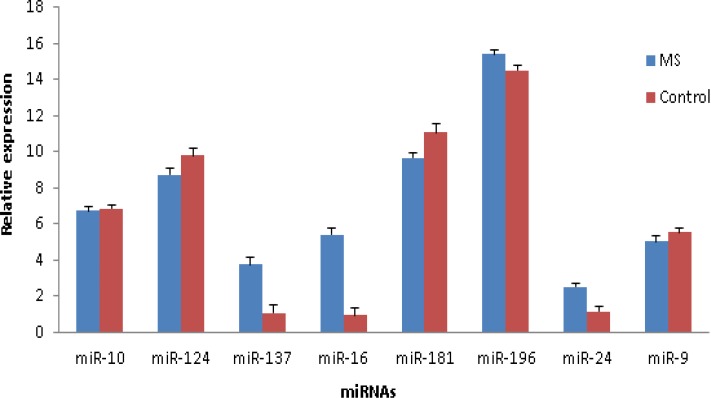
Bar chart of real-time PCR for 8 selected MS related miRNAs. Real-time PCR was performed on serum samples of MS patients and controls. The results were normalized based on the mean of delta CT values for each miRNAs in MS and control samples

To validate our bioinformatics analysis results, eight out of 27 miRNAs were selected for the experimental phase based on both a high number of their uniquely regulated targets and evidences from literature (Table 2).Three of the eight (has-miR-24, has-miR-10 and has-miR-137) are novel and remainder have been previously reported. The expression levels of the eight miRNAs were measured in serum samples from 33 MS patients and 30 healthy controls using real-time PCR. The expression results of miR-16, miR-24, miR-137 (*p*<0.01) and miR-181 (*p*<0.05) were significantly deregulated in MS patients in comparison with the controls. However, the expression level of miR-196, miR-9, miR-10 and miR-124 were not changed significantly in all MS patients (Fig. 5); miR-196 was significantly up regulated (2.24, *p*<0.05) in RRMS patients compared with the control and miR-9 was significantly down regulated(-2.39, *p*<0.05)in PPMS patients (*p*<0.05).

**Table 2:** Summary of potential MS miRNAs biomarkers selected for real-time PCR experiment

**candidate MS miRNA biomarker**	**No. of MS specific targets**	**Biological history**
miR-124	10	Regulate axon guidance [24].
miR-16	8	Control Tregs function [25].
miR-137	2	Regulatory roles in neural development with a potential as a candidate biomarker in neurological disorders [26].
miR-196	1	Regulator of neurological and immunological pathways [27].
miR-9	4	Regulate axon guidance [24].
miR-10	3	Regulator of neurological and immunological pathways [27].
miR-24	4	Regulatory effects on Th1 by targeting INFγ [28].
miR-181	1	B-cell development, regulation of the immune system, neurodegenerative regulatory and neuron protective functions [29].

**Figure 6:**
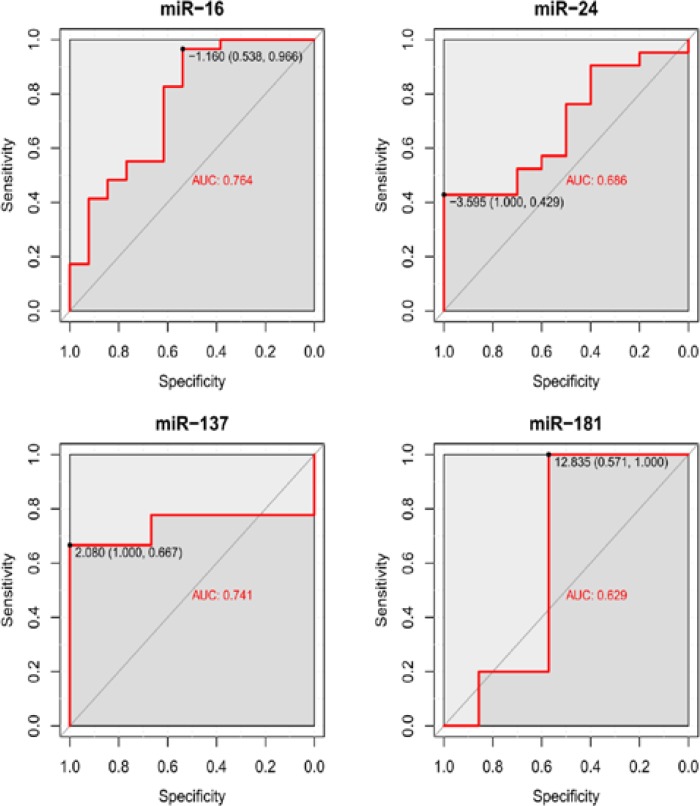
ROC curve analysis for all misregulated miRNAs in MS patients and controls. The diagnostic potential of 4 misregulated miRNAs was evaluated

Receiver Operating Characteristics (ROC) curve analysis was performed to evaluate the diagnostic potential of four significant differentially expressed miRNAs. Briefly, Area under the curve (AUC) was observed for miR-16 (0.76, *p*<0.05), miR-24(0.68,* p*<0.05) miR-137 (0.74, *p*<0.05) and miR-181 (0.62, *p*<0.05) (Fig. 6). In order to understand which of these four miRNAs are novel we considered publically available datasets miR2Disease (www.mir2disease.org) and phenomir (mips.helmholtz-muenchen.de/phenomir) and found that miR-137 and miR-24 were not reported as MS related miRNAs previously. The correlation between EDSS was also examined for significant differentially expressed miRNAs in the MS patients and found the significant correlation between EDSS and the expression level of miR24(ρ=-0.39, *p*<0.01) and miR181 (ρ=-0.33, *p*<0.01).This result proved the negative correlation between EDSS and the expression level of miR-24 and miR-181from the MS patients. This significant inverse correlation allows us to conclude that these two miRNAs have a notable role in disease progression. Another conclusion from the above results is that miR-137 and miR-24 are two novel MS candidate miRNA biomarkers. Also, the deregulation patterns of the four miRNAs together, have the potential to serve as MS related candidate biomarkers.

## DISCUSSION

**Figure 7:**
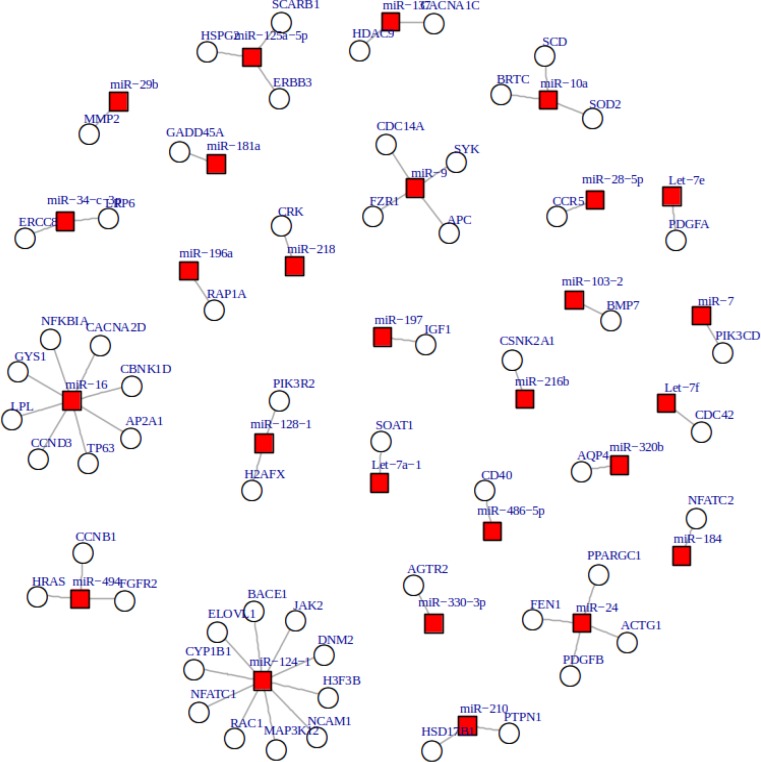
putative mRNA–miRNAs network in MS. Network organization of miRNA and mRNA molecule associations. The network was constructed based on 59 targets with the most significant co-interactions of their related proteins and their 27 relevant miRNAs. Red squares: miRNAs, blue circles: mRNAs

Drawing a good conclusion from the research papers which explored MS related miRNAs is challenging due to variation of studies carried out, i.e. level of study (high or low throughput), source of samples and clinical subtypes used to select the differentially expressed miRNAs. In order to overcome this, a more holistic approach should be used. This requires a comprehensive and multi-staged integrative analysis allowing for a better understanding of this level of complexity. Therefore, we developed a new bioinformatics strategy combining of multi-staged integrative and systematic data analysis. In order to investigate more MS-specific miRNAs, a primary network consisting of the miRNAs and their validated mRNA targets were constructed on the basis of the collected miRNAs. MiRNAs with uniquely regulated targets were scored according to the number of their uniquely regulated targets and the zero scored ones were then removed. This was based on the hypothesis outlined by Zhang et al., in which they propose that if one miRNA has more uniquely regulated targets then it has more potential to be a specific biomarker [23]. To identify the mRNA targets specific to MS, targets were cross-examined with a merged MS related transcriptome dataset as a novel strategy. The resulting common targets are both uniquely regulated and specific to MS. KEGG pathway analysis and protein co-interaction consideration contributed to the design of a bi-layer co-regulatory MS related network model (Fig. 7). The experimental results were also used to verify the network model.

The maximum number of targets belongs to miR-124 (RAC1, MAP3K12, H3F3B, ELOVL1, NCAM1, JAK2, CYP1B1, DNM2, NFATC1 and BACE1), miR-16 (CCND3, TP63, NFKBIA, LPL, GYS1, CACNA2D, AP2A1 and CBNK1D), miR-24 (PDGFB, PPARGC1, ACTG1, FEN1) and miR-9 (CDC14A, FZR1, SYK, APC) in the final co-regulatory network (altogether 22 out of 59 targets). Among these four miRNAs, miR-16 and miR-24 showed significant up regulation in the real-time PCR assays and miR-24 also indicated a good pearson correlation with EDSS and confirmed its role as a novel candidate biomarker. NFKBIA found to be one of the MS specific targets of miR-16. Previously it has been concluded that variations in the promoter region of NFKBIA may be a risk factor of PPMS phenotype [30]. There are many evidences that emphasize the interaction between miR-16 and AU rich elements (AREs) at the 3' UTR of TNF-α and its role is crucial for ARE-mediated mRNA degradation [31]. It has been confirmed that miR-16 has a potential to be a robust biomarker for early detection of MS [13]. Overexpression of miR-16 in T cells of MS patients in comparison with healthy donors has been previously reported [25]. INFγ is responsible for differentiation of CD4+ T cells into Th1 and is also secreted by these effector cells. MiR-24 targets INFγ in Th1 cells and has the potential to be an important regulator of many autoimmune diseases such as MS [28]. It has been suggested that PDGF which is one of the miR-24 targets in the constructed network, has a significant role in the prevention of relapse in patients.MiR-137 (another novel biomarker) represented the significant down regulation in the experimental phase [32]. Recently it has been propounded that miR-137 has a great potential to be a diagnostic and prognostic biomarker for many neuroglial disorders [26]. Here we foundHDAC9 and CACNA1Cas MS related targetsof miR-137 in the final regulatory network. Histone deacetylases (HDACs) are the therapeutic targets in some neurological disorders such as MS [33].One study on an EAE model has suggested that calcium influx through voltage- gated calcium channels (CACN) may have a role in inflammatory neurodegeneration disorders[34]. Another misregulated miRNA was miR-181; which showed a significant Pearson correlation with EDSS. MiR-181 is involved in B-cell development in brain and retina, and plays a role in regulation of the immune system and also some neurodegenerative regulatory and neuron protective functions [29]. Together, these findings may help to better understand the mechanism underlying the MS and find candidate miRNA biomarkers for MS.

The experimental results validated our proposed bioinformatics strategy. Our strategy was used to find novel candidate miRNA biomarker for MS. Construction of the MS related miRNA-mRNA regulatory network helped to reveal disease related pathways. The results suggest that miR-24 and miR-137 can be used as novel candidate biomarkers for MS. The next stage is to further validate these findings using a larger cohorts and datasets.
